# A Facile Surface Modification Strategy for Antibody Immobilization on 3D-Printed Surfaces

**DOI:** 10.3390/bios15040211

**Published:** 2025-03-25

**Authors:** Brandi Binkley, Peng Li

**Affiliations:** Department of Chemistry, West Virginia University, Morgantown, WV 26506, USA; bb00011@mix.wvu.edu

**Keywords:** surface modification, 3D printing, ELISA

## Abstract

3D-printed microdevices have become increasingly important to the advancement of point-of-care (POC) immunoassays. Despite its great potential, using 3D-printed surfaces on the solid support for immunorecognition has been limited due to the non-ideal adsorption properties for many photocurable resins. In this work, we report a simple surface modification protocol that works for diverse commercial photocurable resins, improving ELISAs performed directly on 3D-printed devices. This surface modification strategy involves surface activation via air plasma followed by the one-step incubation of GLYMO-labeled streptavidin. We successfully immobilized biotinylated anti-activin A antibodies on the 3D-printed surfaces and performed the complete ELISA protocol on the 3D-printed surfaces. We demonstrated that this protocol achieved an improved performance over passive adsorption for ELISAs. The present method is also compatible with diverse commercial resins and works with both microwells and microchannels. Finally, this method demonstrated a comparable limit of detection to the ELISA performed using commercial microwells. We believe the simplicity and broad compatibility of the present surface modification strategy will facilitate the development of 3D-printed POC ELISA devices.

## 1. Introduction

Three-dimensional printing has become a popular tool for making microfluidic devices in recent years. The use of a 3D printer allows researchers to easily design devices with their desired geometries and structures and then print them right in the same lab. This process significantly decreases the cost and skill barrier of creating customized devices, as well as shortens the device iteration process. To date, 3D-printed microdevices have been reported for many applications, including droplet separation, generation, and sorting, microfluidics, cell manipulation, and multiple forms of detection/diagnostics [1,2,3,4,5,6,7,8,9,10]. Some applications have combined multiple components, such as 3D-printed microfluidic devices for electrochemical detection through the fabrication of devices that incorporate electrodes [11,12,13,14,15].

One emerging area is the utilization of the flexibility of 3D-printed structures to achieve sophisticated fluid manipulation without the need for complex external equipment, which has great potential to advance the point-of-care (POC) testing of various biomarkers. Juncker and coworkers reported a 3D-printed microfluidic chain reaction (MCR) device to achieve programmable fluid operations using paper-driven capillary flow [16]. They demonstrated complete ELISA operations with the MCR device, and the reaction signals were detected using a test strip. He et al. reported 3D-printed microfluidic composable plates (cPlates) for multiplexed ELISA assays that achieved the simultaneous handling of 48 individual wells via the assembly and disassembly of the 3D-printed plates. They demonstrated the simultaneous quantification of four protein biomarkers using a magnetic bead-based ELISA without the need for robotics [17]. Others have used the standard 96-well plate wells along with their 3D-printed devices. Bauer and Kulinsky applied 3D printing to fabricate 3D scaffolds for commercial ELISA wells and fluid paths, which enables the automatic distribution of detection reagents to ELISA wells [18]. Kadimisetty et al. reported a 3D-printed microfluidic device for automatic fluid operations. They combined this device with a single-wall carbon nanotube-modified pyrolytic graphite sheet for the simultaneous detection of eight protein biomarkers for prostate cancer [19]. Ukita et al. employed 3D printing to fabricate centrifugal microfluidic devices for automatic ELISAs. The solution was added to the microfluidic chamber, with centrifugation and spinning performed during the incubation steps [20]. McCallen et al. employed 3D-printed pillars as the solid support for the ELISA. Instead of manipulating fluids, a complete ELISA protocol was achieved by controlling the movement of the pillar arrays in and out of a standard 96-well plate [21]. While these 3D-printed devices have enabled new schemes for performing ELISAs, they often utilize external solid supports for the immunorecognition reactions, including well plates, magnetic beads, and test strips. Performing ELISA reactions directly on 3D-printed surfaces has the potential to further reduce the complexity of device fabrication and fluid manipulation, thereby enabling new 3D-printed ELISA devices for POC applications.

To date, there are numerous studies on the modification of 3D-printed materials for various applications, including implants in the medical field, improved biocompatibility, preventing biofilm formation, improved longevity of the 3D-printed device, and enhanced cell adhesion [22,23,24,25,26]. Some modifications have been made to the printing material before the 3D printing process for biosensing. These applications have used graphene–PLA material for a 3D-printed electrode that can be used in biosensing applications. After printing, different modifications can be made to the electrode surface, such as activation or modification with different enzymes or nanoparticles [27,28,29]. Other researchers have looked at treating the 3D-printed electrode surface through mechanical polishing and electrochemical treatment with NaOH [30,31]. However, studies on surface modification for ELISAs have been limited. The majority of the existing works rely on passive adsorption for immobilizing antibodies on 3D-printed surfaces [21], particularly on thermoplastics, including PLA (Polylactic Acid) and ABS (Acrylonitrile Butadiene Styrene). Singh et al. employed chemical etching for ABS materials to enhance their hydrophilicity, which improves the adsorption of antigens on the surface for ELISAs [32]. To achieve higher printing resolutions and complex microstructures, photopolymerization-based printing is needed. However, for photocurable resins, passive adsorption is not ideal for the ELISA performance, as shown in the manuscript. Sharafeldin et al. fabricated a thin layer of chitosan on the surfaces of 3D-printed devices with photocurable resins. The amine groups were then activated by adding a glutaraldehyde solution to the surface before immobilizing capture antibodies for the ELISA [33]. To date, there is no report of using the plasma-based method for photocurable resins for 3D-printed ELISA devices.

**Figure 1:**
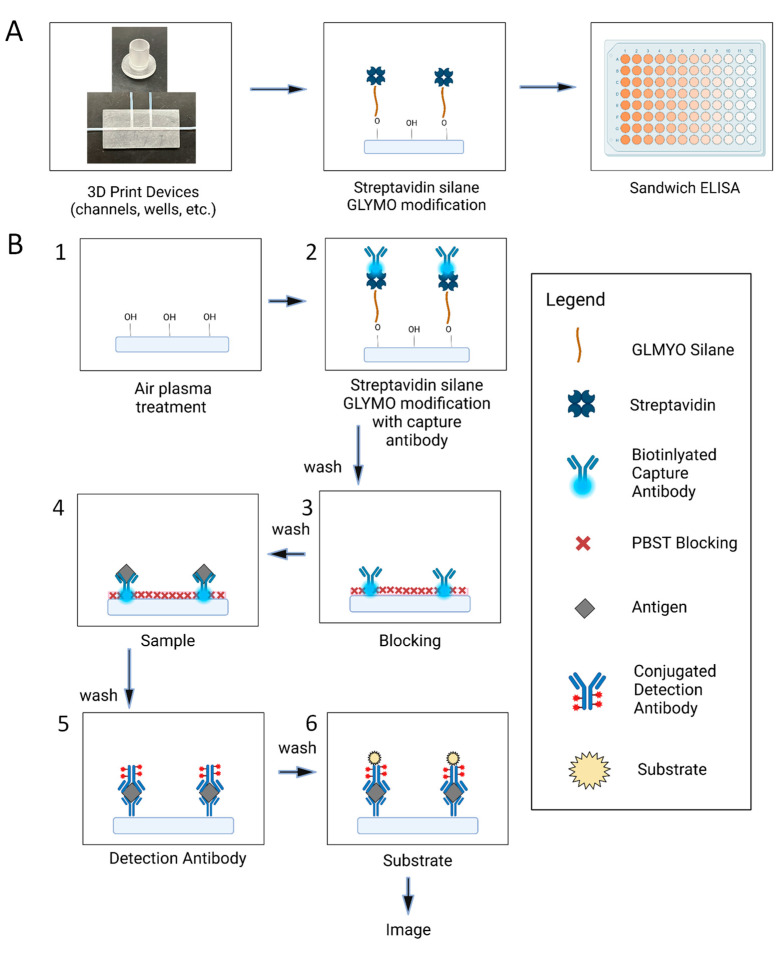
(**A**) overview of the key steps of the procedure from the when the devices are made until the fluorescent readout (**B**) detailed steps of the ELISA protocol that is performed on the various 3D printed surfaces. 1. Air plasma treatment where hydroxyls are added to the surface 2. GLYMO modification of the streptavidin that binds to the hydroxyls along with the addition of the capture antibody 3. 2% BSA solution was added to block any remaining sites on the surface 4. Samples of varying concentration were added to bind to the capture antibody 5. Detection antibody was added to bind to the existing capture antibody sample complexes 6. Substrate was added so that a fluorescent signal would be produced to image.

In this work, we report a new strategy for preparing 3D-printed surfaces for ELISAs (Figure 1A). The surface modification is accomplished through the use of plasma treatment and a (3-glycidyloxpropyl) trimethoxyl-silane (GLYMO) modification. This strategy is simple and quick to perform under mild conditions. Plasma treatment can be used to modify the surface chemistries of a variety of materials by introducing functional groups though plasma gases. In this case, air plasma introduces hydroxyl groups on the surface and enhances the hydrophilicity of the surface, which facilitate the immobilization of streptavidin on the surface [34]. We demonstrated that 3D-printed wells modified with the present strategy achieved a similar performance to that of commercial ELISA wells. This method is compatible with various commercial photocurable resins and works for both open-well systems and closed-microchannel devices. We believe that the present surface modification strategy will enable novel 3D-printed POC ELISA devices and facilitate their adoption by the broader community.

## 2. Materials and Methods

### 2.1. Materials and Reagents

Different 3D printing commercial resins were purchased for direct use without modification: Nanoclear was purchased from FunToDo (Kotka, Finland), Dentrifix was purchased from Resine-3D (Bruay-sur-I’Escaut, France), and Conjure Rigid was purchased from Chitu Systems through Amazon (Seattle, WA, USA). Phosphate-buffered saline (PBS) was purchased from Corning (Corning, NY, USA), while phosphate-buffered silane with Tween (PBST) was bought from Thermo Fisher (Waltham, MA, USA). The commercial ELISA kit and reagents used were obtained from rndystems (Minneapolis, MN, USA). This was a human, mouse, and rat activin A quantikine ELISA kit. The substrate used was the QuantaRed Enhanced Chemifluorescent HRP substrate kit and was purchased from Thermo Fisher. Streptavidin was also purchased from the same vendor, Thermo Fisher. (3-glycidyloxpropyl)trimethoxyl-silane (GLYMO) was bought from TCI (Tokyo Chemical Industry, Tokyo, Japan).

### 2.2. Device Design and Fabrication

All of the 3D-printed devices were designed on SolidWorks 2017, a computer software program that is used for 3D computer-aided design (CAD) [35]. The detailed dimensions of each device are as follows: The small open wells are 1.5 mm in depth and 2 mm in diameter (Figure S1), while the larger open wells are 11 mm in depth and 7 mm in diameter (Figure S2). There are also medium open wells that are 3 mm in diameter and 2.25 mm in depth (Figure S3). The closed-channel device has a channel that is 40 mm long and 1.3 mm in diameter. This channel is enclosed in a device that is 40 mm long in total, 20 mm wide, and 3 mm in height (Figure S4). Each design was fabricated using LCD resin 3D printing. The printer used was a Phrozen Sonic 8K (Phrozen Technology, Hsinchu City, Taiwan), which has a 7.1-inch LCD screen and an XY resolution of 28 μm [36]. Multiple 3D printing resins were tested throughout the experimentation process, but the one that was chosen for the majority of the paper’s work was Chitu System’s Conjure Rigid. This is a high-precision resin that is more hydrophobic in nature compared to many of the resins typically used in 3D printing, and it has a hardness shore D of 80–90, which indicates that it is on the more rigid side of resins [37]. The exposure time and bottom exposure time were the settings that were optimized on the 3D print for each device design and resin. For the large wells, the exposure time was 2.5 ms, while the bottom exposure time was 30 ms. The small and medium wells had an exposure time of 5 ms and a bottom exposure time of 10 ms. Closed-channel device printer settings were similar, with exposure and bottom exposure times of 3 ms and 10 ms, respectively.

### 2.3. Surface Preparation and Streptavidin Immobilization

First, air plasma treatment was applied to the 3D-printed device. This is performed using a Harrick Plasma Plasma Cleaner PDC-001 (Harrick Plasma, Ithaca, NY, USA), with the surface results shown in Figure 1B (step 1). The vacuum runs for 5 min before the UV light is turned on high for a set amount of time depending on the device design. Next, a stock solution of 10 mM GLYMO in PBS buffer was prepared by weighing out 660 mg of GLYMO and diluting it with PBS buffer to 1 mL. The stock was then diluted further to 4.8 × 10^−7^ M [38]. Immediately after the plasma treatment, this GLYMO solution was mixed with streptavidin and added to the surfaces of 3D-printed devices (Figure 1B, step 2). The streptavidin GLYMO mixture is made by taking 198 μL of the previously made stock and adding 2 μL of 1 mg/mL streptavidin. This makes the concentration of the streptavidin protein 0.1 mg/mL, which has previously been used in immobilization studies [39]. This can be adjusted depending on the total volume needed. The devices can then be stored at 4 °C before the ELISA.

### 2.4. ELISA Protocol

The ELISA protocol for each experiment followed the general steps of the commercial ELISA kit [40]. All incubation steps were completed at room temperature. Biotinylated activin A antibody was added to the wells and incubated for 15 min. After washing, 2% BSA was used to block the wells for 45 min (Figure 1B, step 3). Different concentrations of activin A were then added to the wells and incubated for 3 h (Figure 1B, step 4). After washing, HRP-conjugated detection antibodies were added and incubated for 1 h (Figure 1B, step 5). Finally, fluorescence signals were generated using the QuantaRed Enhanced Chemifluorescent HRP Substrate Kit (Thermo Fisher, Waltham, MA, USA) (Figure 1B, step 6). The working solution was prepared by mixing QuantaRed Stable Peroxide Solution, QuantaRed Enhancer Solution, and QuantaRed ADHP concentrate with a volume ratio of 50:50:1. The signals were then measured using an inverted fluorescence microscope (Zeiss Axiovert 200m, ZEISS, Oberkochen, Germany) with a sCMOS camera (Andor Zyla 4.2+, Andor Technology Ltd., Belfast, Northern Ireland, UK). The reagent volume used was dependent on the well size being used. For the large wells (diameter: 7 mm), a reagent volume of 200 μL was used. For the medium wells (diameter: 3 mm), a reagent volume of 30 μL was used. For the small wells (diameter: 2 mm), a reagent volume of 5 μL was used.

## 3. Results and Discussion

### 3.1. Surface Modification Principle and Procedure

Three-dimensional-printed microdevices have utilized diverse photocurable resins as their channel materials. Unlike classic plastic surfaces (e.g., polystyrene), the properties of these photocurable resins may not facilitate the adsorption of capture antibodies or antigens on the surface. To achieve the robust immobilization of capture antibodies on photocurable resins, we designed a two-step surface modification strategy to prepare the surfaces of 3D-printed devices for ELISAs (Figure 1B). The first step is the activation of the 3D-printed surfaces using air plasma treatment. This step introduces free hydroxyl groups on the surface and allows for the covalent attachment of silanes to the surface. Although the recipes of the commercial resins used here are proprietary, they should be mostly based on acrylate or methacrylate groups. The major effects of plasma treatment include the introduction of oxygen-containing functional groups (e.g., –OH), the removal of organic contaminants, and a minor increase in the surface roughness. These effects facilitate the immobilization of biomolecules on 3D-printed surfaces [41,42,43]. In our experiments, the activation of photocurable resin surfaces via air plasma was confirmed by the decrease in the contact angle for water droplets on the 3D-printed surface after treatment, which can be seen in Figure S5. In the second step, we utilized (3-glycidyloxpropyl) trimethoxyl-silane (GLYMO) to immobilize proteins on the surfaces. GLYMO can be attached to surfaces via the hydroxyl group, and the epoxy ring on the other end allows for the covalent labeling of proteins via primary amines. It should be noted that when the GLYMO is mixed with PBS buffer, the trimethoxy-silyl groups will be hydrolyzed to form silanol groups. These silanol groups are significantly less reactive to the amines in streptavidin compared to the epoxy group in GLYMO. In addition, the short duration between mixing GLYMO and streptavidin and applying the solution to the surface further limits the extent of unwanted side reactions. In this study, we first mixed GLYMO and streptavidin in the solution and then added the solution to the surfaces of 3D-printed devices. The immobilized streptavidin enables the subsequent immobilization of biotinylated antibodies, which are commonly used in many commercial ELISA kits. It is also possible to mix GLYMO with capture antibodies directly if biotinylated antibodies are not available. This procedure is easy to carry out without the need for harsh reaction conditions or special operation procedures.

### 3.2. Demonstration of ELISA

**Figure 2:**
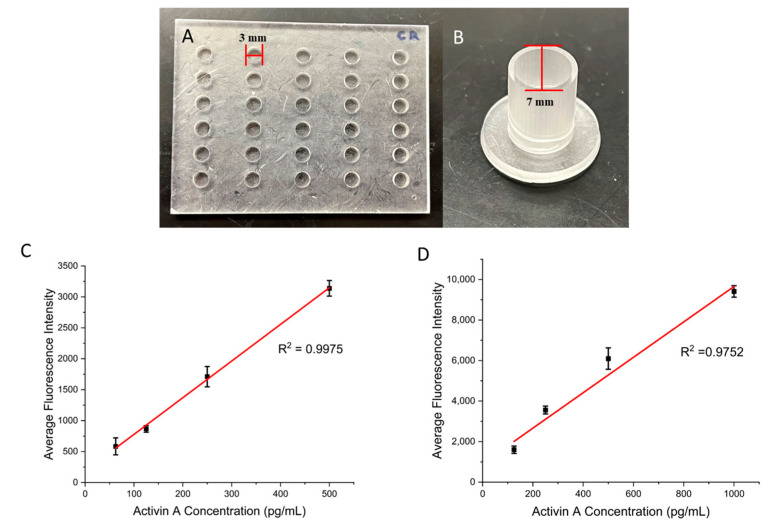
(**A**) Three-dimensional-printed medium wells that are 3 mm in diameter and 2.25 mm in height. (**B**) Three-dimensional-printed large wells that are 7 mm in diameter and 11 mm in height. (**C**) Calibration curve for the medium wells with a range of Activin A concentrations. (**D**) Calibration curve for the large wells with a range of Activin A concentrations. Error bars represent standard deviations of three trials.

To test the performance of the present surface modification strategy, we printed ELISA wells with two different sizes: medium-sized wells that were 3 mm in diameter and 2.25 mm in height, along with larger-sized wells that were 7 mm in diameter and 11 mm in height (Figure 2A,B). A commercial photocurable resin, Conjure Rigid, was used for printing. This resin is known for its high precision, transparent color, strength, and ability to be used for functional 3D parts. The ELISA reagents were used from a commercial kit for detecting activin A. The biotinylated anti-activin A antibody was immobilized on the 3D-printed wells following the steps described in the previous section. Figure 2C,D shows the calibration curves of the ELISA assay for different sizes of wells. Values for the calibration curves are available in Table S1. In both cases, there is a linear relationship between the fluorescence signals and the activin concentrations: from 62.5 pg/mL to 500 pg/mL for the medium wells and from 125 pg/mL to 1000 pg/mL for the 3D-printed wells, with R^2^ values of 0.9975 and 0.9752, respectively, indicating the effectiveness of the present coating strategy. The present coating strategy works for diverse sizes of wells, and we expect a minimal impact of the well dimensions on the coating outcome. However, for optimal ELISA results, the dimensions of the wells need to be optimized for the desired application. For example, larger wells could offer improved sensitivity at the expense of higher sample consumption.

**Figure 3:**
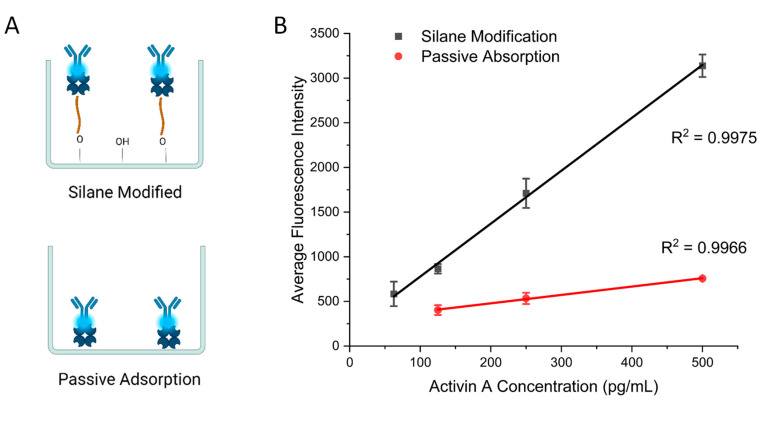
(**A**) Schematic of two antibody immobilization methods. (**B**) Calibration curves comparing the silane modification protocol and passive adsorption with activin A ELISA. Error bars represent standard deviations of three trials.

To demonstrate the necessity of surface modification, we compared the present surface modification strategy with passive adsorption for ELISAs. The first group of wells were prepared based on the protocol listed above. The second group of wells were prepared by incubating the streptavidin solution directly with the 3D-printed wells (Figure 3A). The rest of the protocol after this step was identical. Both groups were then used to perform ELISAs for activin A with concentrations from 62.5 pg/mL to 500 pg/mL in the medium wells with a diameter of 3 mm and height of 2.25 mm. Figure 3B shows the calibration curves obtained using the two types of surface modification. Values for the calibration curves are available in Table S2. Both groups achieved a linear relationship between the activin concentration and fluorescence signal. However, the slope of the passive-adsorption group of 0.94 is significantly smaller than the one obtained with the modified surface of 5.92, which indicates the improvement in the assay sensitivity with the modified surface over adsorption. We also compared the limit of detection (LOD) for the two groups. The modified wells achieved an LOD of 34 pg/mL, whereas the unmodified wells showed an LOD of 102 pg/mL. These results demonstrate the need for improved surface modification for performing ELISAs using 3D-printed devices.

**Figure 4:**
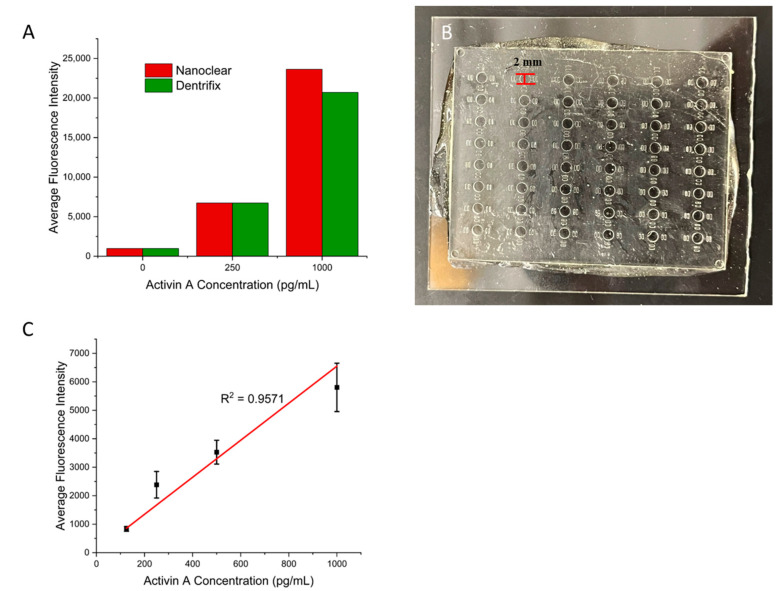
(**A**) A bar graph showing the results of the ELISA for activin A performed using different commercial resins. (**B**) Three-dimensional-printed small wells that are 2 mm in diameter and 1.5 mm in height. (**C**) Calibration curve for the small wells with ranging Activin A concentrations. Error bars represent standard deviations of three trials.

Next, we examined whether this protocol works for other commercial resins. In addition to the Conjure Rigid resin, we also tested two other clear 3D printing resins: Nanoclear and Dentrifix. Nanoclear is known for having high resolution and clarity, which are ideal for microfluidic devices. Dentrifix is also a clear resin that is often used for dental applications. First, we printed wells that were the same size as those on a traditional 96-well plate. They were 11 mm in height and 7 mm in diameter. The ELISA results are shown in Figure 4A, indicating successful ELISAs for both resins. These results support the broad applicability of the present surface modifications for diverse commercial photocurable resins. In addition, we printed small wells that were 2 mm in diameter and 1.5 mm in height to test the ability to use the protocol with smaller volumes using Nanoclear (Figure 4B). We tested activin A concentrations from 125 pg/mL to 1000 pg/mL. Again, a linear relationship between the concentration and the fluorescence signal was achieved (Figure 4C). Values for the calibration curve are available in Table S3. This result demonstrated that the present protocol for small structures also works for small structures that have been used in microfluidic cPlate methods [17]. It should be noted that the results obtained with small wells showed larger variations across replicates. This is due to the small size of the well, making standard pipette operations more challenging.

### 3.3. Surface Modification in 3D-Printed Microchannels

**Figure 5:**
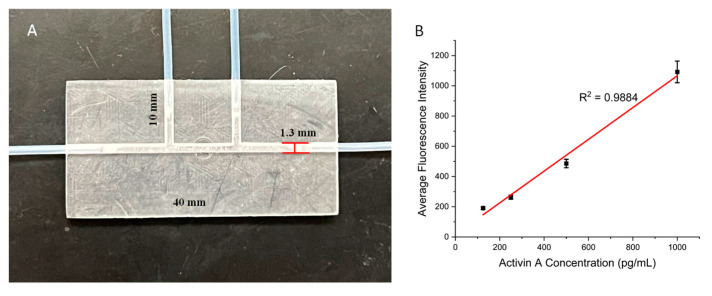
(**A**) Three-dimensional-printed channel device with a main channel for performing on-chip ELISAs and two side channels for loading surface modification solutions. (**B**) Calibration curve for the channel device with a range of Activin A concentrations. Error bars represent standard deviations of three trials.

The above results showed that the present surface modification strategy works for 3D-printed open wells. Many 3D-printed microdevices also involve closed microchannels, which facilitate the manipulation of fluids at the microscale. Here, we examined whether the present strategy also works for closed microchannels. We printed a channel device that was 40 mm in length, 20 mm in width, and 3 mm in height. The device had a straight channel with a diameter of 1.3 mm going across the entire length of the device. There were also two side channels with the same diameter that connected to this channel in the middle of the device, as they had a length of 10 mm. These sized openings allowed for the tubing to be directly inserted into the channel (Figure 5A). The major concern for the microchannel surface modification was the effectiveness of the air plasma treatment. We first tested it using the protocol above, and the results showed low signals, with all concentrations having similar fluorescent intensities to those of the blank control. We then found that by simply extending the plasma treatment time from 5 min to 30 min, a successful ELISA could be achieved. We tested the ELISA in the microchannel using activin A. For the streptavidin immobilization, the streptavidin–GLYMO solution was loaded from one of the side channels and incubated for 45 min after the plasma treatment, with the solution being refreshed every 15 min. The inlet and outlet of the main channel were closed in this step using syringes. For the following ELISA steps, the side channels were closed, and all the reagents and washing buffers were loaded from the main channel inlet using a syringe. Figure 5B shows the calibration curve of the microchannel-based ELISA for activin A. Values for the calibration curves are available in Table S4. For activin concentrations from 125 pg/mL to 1000 pg/mL, a linear calibration curve was obtained with an R^2^ value of 0.9884. This result demonstrated that the present surface modification strategy can also be applied to 3D-printed microchannels. Here, we tested channels with a 1.3 mm diameter with success. For smaller channels, an extended plasma treatment time may be needed to ensure optimal surface conditions for protein immobilization.

### 3.4. Comparison with Commercial ELISA Well Plates

**Figure 6:**
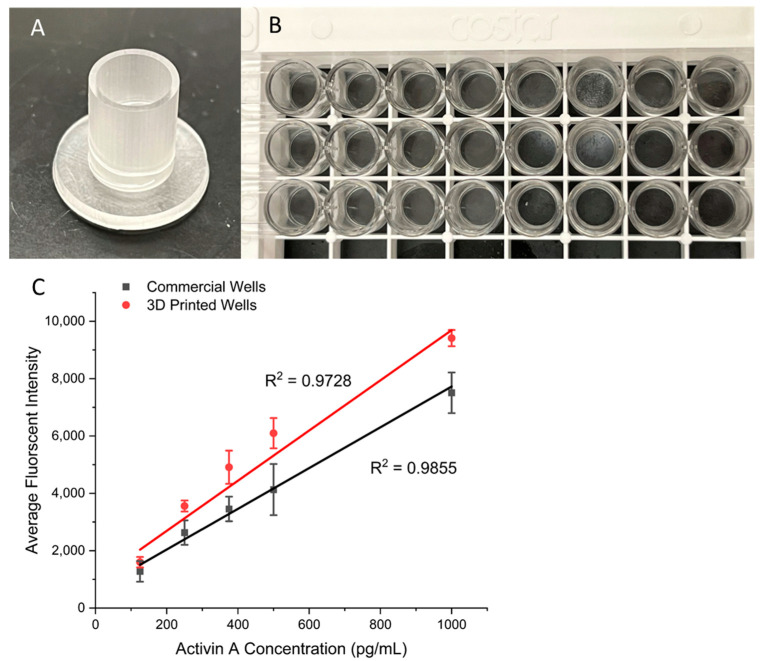
(**A**) Three-dimensional-printed well that has the same size as that of a standard well of a 96-well plate. (**B**) A commercial standard 96-well plate for ELISAs. (**C**) Calibration curves comparing the commercial kit to the 3D-printed wells with ranging Activin A concentrations. Error bars represent standard deviations of three trials.

Finally, we validated the performance of the present surface modification strategy for ELISAs with wells from a commercial kit. For the comparison to be fair, the same samples, reagents, and protocol steps were used. The only difference was what the ELISAs were run on. One set was run on the 96-well plate provided in the kit and the other on the 3D-printed surface-coated wells of the same size (Figure 6A,B). A series of concentrations of Activin A were tested so that the fluorescent intensities could be compared among the two, along with other key measurements, such as the relative error and standard deviation. The results can be seen in Figure 6C, which shows that the 3D-printed wells had a slightly higher slope of 8.75 than the commercial ELISA kit’s slope of 7.11, indicating a slight increase in sensitivity for the 3D-printed device. For the LOD comparison, the 3D-printed wells achieved an LOD of 6 pg/mL, whereas the commercial kit achieved an LOD of 7 pg/mL. Values for the calibration curves are available in Table S5. Overall, the results show that by using the surface modification protocol reported here, 3D-printed devices can achieve a similar level of performance to that of the plastic wells used in commercial ELISA kits while providing flexibility in the design of various fluid manipulation structures. We believe that this simple modification strategy will facilitate the adoption and development of 3D-printed devices for various bioassays.

## 4. Conclusions

In summary, we have developed a surface modification coating strategy that can be applied to 3D-printed surfaces for the immobilization of antibodies for ELISAs. Air plasma treatment for surface modification allows for a simple approach to optimizing the surface chemistries of 3D-printed resins. The air plasma treatment length varies depending on the device design, as it may take longer to reach all the intended surfaces, especially if they are not fully open, like a channel. We successfully applied the surface modification coating strategy to different resins, open wells of varying sizes, and a closed-channel device. This modification strategy is simple to perform and can be used for various 3D printing materials. It is important to note that the well and channel sizes impact the sensitivity and detectability in multiple aspects, as the size does not only affect the surface modification but also the volume and operation. Our results indicate that the surface modification was successful no matter the dimensions, but as the well size became smaller, the performance was reduced due to the mentioned factors. Future work will focus on combining this surface modification protocol with improved passivation strategies to further enhance the sensitivity of ELISAs. In addition, we anticipate that this method will also enable novel 3D-printed microdevices for POC ELISAs with improved capabilities and reduced assay complexity.

## Data Availability

The datasets for the ELISA experiments are provided with the Supporting Information.

## Supplementary Materials

The following supporting information can be downloaded at https://www.mdpi.com/article/10.3390/bios15040211/s1. Table S1: ELISA data points for Figure 2C,D; Table S2: ELISA data points for Figure 3B; Table S3: ELISA data points for Figure 4C; Table S4: ELISA data points for Figure 5B; Table S5: ELISA data points for Figure 6C; Figure S1: Three-dimensional-printed device with small-well design and dimensions. Figure S2: Three-dimensional-printed large-well device design and dimensions; Figure S3: Three-dimensional-printed medium-well plate device design and dimensions; Figure S4: Three-dimensional-printed channel device design and dimensions; Figure S5: Contact angle study.

## Abbreviations

The following abbreviations are used in this manuscript:

ELISA	Enzyme-linked immunosorbent assay
POC	Point of care
GLYMO	(3-glycidyloxpropyl) trimethoxyl-silane

## References

[1] Berlanda S.F., Breitfeld M., Dietsche C.L., Dittrich P.S. (2021). Recent Advances in Microfluidic Technology for Bioanalysis and Diagnostics. Anal. Chem..

[2] Ali M.A., Hu C., Yttri E.A., Panat R. (2022). Recent Advances in 3D Printing of Biomedical Sensing Devices. Adv. Funct. Mater..

[3] Chan H.N., Tan M.J.A., Wu H. (2017). Point-of-Care Testing: Applications of 3D Printing. Lab Chip.

[4] Duarte L.C., Figueredo F., Chagas C.L.S., Cortón E., Coltro W.K.T. (2024). A Review of the Recent Achievements and Future Trends on 3D Printed Microfluidic Devices for Bioanalytical Applications. Anal. Chim. Acta.

[5] He Y., Wu Y., Fu J., Gao Q., Qiu J. (2016). Developments of 3D Printing Microfluidics and Applications in Chemistry and Biology: A Review. Electroanalysis.

[6] Lambert A., Valiulis S., Cheng Q. (2018). Advances in Optical Sensing and Bioanalysis Enabled by 3D Printing. ACS Sens..

[7] Liu C., Staples R., Gómez-Cerezo M.N., Ivanovski S., Han P. (2023). Emerging Technologies of Three-Dimensional Printing and Mobile Health in COVID-19 Immunity and Regenerative Dentistry. Tissue Eng. Part C Methods.

[8] Nyenhuis J., Heuer C., Bahnemann J. (2024). 3D Printing in Biocatalysis and Biosensing: From General Concepts to Practical Applications. Chem. Asian J..

[9] Prabhakar P., Sen R.K., Dwivedi N., Khan R., Solanki P.R., Srivastava A.K., Dhand C. (2021). 3D-Printed Microfluidics and Potential Biomedical Applications. Front. Nanotechnol..

[10] Remaggi G., Zaccarelli A., Elviri L. (2022). 3D Printing Technologies in Biosensors Production: Recent Developments. Chemosensors.

[11] Kalinke C., Wosgrau V., Oliveira P.R., Oliveira G.A., Martins G., Mangrich A.S., Bergamini M.F., Marcolino-Junior L.H. (2019). Green Method for Glucose Determination Using Microfluidic Device with a Non-Enzymatic Sensor Based on Nickel Oxyhydroxide Supported at Activated Biochar. Talanta.

[12] Caetano F.R., Carneiro E.A., Agustini D., Figueiredo-Filho L.C.S., Banks C.E., Bergamini M.F., Marcolino-Junior L.H. (2018). Combination of Electrochemical Biosensor and Textile Threads: A Microfluidic Device for Phenol Determination in Tap Water. Biosens. Bioelectron..

[13] de C., Costa B.M., Griveau S., Bedioui F., Orlye F.D., Da Silva J.A.F., Varenne A. (2022). Stereolithography Based 3D-Printed Microfluidic Device with Integrated Electrochemical Detection. Electrochim. Acta.

[14] Carvalho R.M., Ferreira V.S., Lucca B.G. (2021). A Novel All-3D-Printed Thread-Based Microfluidic Device with an Embedded Electrochemical Detector: First Application in Environmental Analysis of Nitrite. Anal. Methods.

[15] Salve M., Mandal A., Amreen K., Pattnaik P.K., Goel S. (2020). Greenly Synthesized Silver Nanoparticles for Supercapacitor and Electrochemical Sensing Applications in a 3D Printed Microfluidic Platform. Microchem. J..

[16] Yafia M., Ymbern O., Olanrewaju A.O., Parandakh A., Sohrabi Kashani A., Renault J., Jin Z., Kim G., Ng A., Juncker D. (2022). Microfluidic Chain Reaction of Structurally Programmed Capillary Flow Events. Nature.

[17] He Z., Huffman J., Curtin K., Garner K.L., Bowdridge E.C., Li X., Nurkiewicz T.R., Li P. (2021). Composable Microfluidic Plates (cPlate): A Simple and Scalable Fluid Manipulation System for Multiplexed Enzyme-Linked Immunosorbent Assay (ELISA). Anal. Chem..

[18] Lim C., Lee Y., Kulinsky L. (2018). Fabrication of a Malaria-Ab ELISA Bioassay Platform with Utilization of Syringe-Based and 3D Printed Assay Automation. Micromachines.

[19] Kadimisetty K., Malla S., Bhalerao K.S., Mosa I.M., Bhakta S., Lee N.H., Rusling J.F. (2018). Automated 3D-Printed Microfluidic Array for Rapid Nanomaterial-Enhanced Detection of Multiple Proteins. Anal. Chem..

[20] Ukita Y., Utsumi Y., Takamura Y. (2016). Direct Digital Manufacturing of a Mini-Centrifuge-Driven Centrifugal Microfluidic Device and Demonstration of a Smartphone-Based Colorimetric Enzyme-Linked Immunosorbent Assay. Anal. Methods.

[21] McCallen J.D., Schaefer A., Lee P., Hing L., Lai S.K. (2017). Stereolithography-Based 3D Printed “Pillar Plates” That Minimizes Fluid Transfers During Enzyme Linked Immunosorbent Assays. Ann. Biomed. Eng..

[22] Salehi S., Tavakoli M., Mirhaj M., Varshosaz J., Labbaf S., Karbasi S., Jafarpour F., Kazemi N., Salehi S., Mehrjoo M. (2023). A 3D Printed Polylactic Acid-Baghdadite Nanocomposite Scaffold Coated with Microporous Chitosan-VEGF for Bone Regeneration Applications. Carbohydr. Polym..

[23] Ni R., Jing Z., Xiong C., Meng D., Wei C., Cai H. (2022). Effect of Micro-Arc Oxidation Surface Modification of 3D-Printed Porous Titanium Alloys on Biological Properties. Ann. Transl. Med..

[24] Imani S.M., Badv M., Shakeri A., Yousefi H., Yip D., Fine C., Didar T.F. (2019). Micropatterned Biofunctional Lubricant-Infused Surfaces Promote Selective Localized Cell Adhesion and Patterning. Lab Chip.

[25] Jaidev L.R., Chatterjee K. (2019). Surface Functionalization of 3D Printed Polymer Scaffolds to Augment Stem Cell Response. Mater. Des..

[26] Saniei H., Mousavi S. (2020). Surface Modification of PLA 3D-Printed Implants by Electrospinning with Enhanced Bioactivity and Cell Affinity. Polymer.

[27] Stefano J.S., Guterres e Silva L.R., Rocha R.G., Brazaca L.C., Richter E.M., Abarza Muñoz R.A., Janegitz B.C. (2022). New Conductive Filament Ready-to-Use for 3D-Printing Electrochemical (Bio)Sensors: Towards the Detection of SARS-CoV-2. Anal. Chim. Acta.

[28] Martins G., Gogola J.L., Budni L.H., Janegitz B.C., Marcolino-Junior L.H., Bergamini M.F. (2021). 3D-Printed Electrode as a New Platform for Electrochemical Immunosensors for Virus Detection. Anal. Chim. Acta.

[29] López Marzo A.M., Mayorga-Martinez C.C., Pumera M. (2020). 3D-Printed Graphene Direct Electron Transfer Enzyme Biosensors. Biosens. Bioelectron..

[30] Rocha D.P., Squissato A.L., Da Silva S.M., Richter E.M., Munoz R.A.A. (2020). Improved Electrochemical Detection of Metals in Biological Samples Using 3D-Printed Electrode: Chemical/Electrochemical Treatment Exposes Carbon-Black Conductive Sites. Electrochim. Acta.

[31] Stefano J.S., Kalinke C., Da Rocha R.G., Rocha D.P., Da Silva V.A.O.P., Bonacin J.A., Angnes L., Richter E.M., Janegitz B.C., Muñoz R.A.A. (2022). Electrochemical (Bio)Sensors Enabled by Fused Deposition Modeling-Based 3D Printing: A Guide to Selecting Designs, Printing Parameters, and Post-Treatment Protocols. Anal. Chem..

[32] Singh H., Shimojima M., Fukushi S., Le Van A., Sugamata M., Yang M. (2015). Increased Sensitivity of 3D-Well Enzyme-Linked Immunosorbent Assay (ELISA) for Infectious Disease Detection Using 3D-Printing Fabrication Technology. Biomed. Mater. Eng..

[33] Sharafeldin M., Kadimisetty K., Bhalerao K.R., Bist I., Jones A., Chen T., Lee N.H., Rusling J.F. (2019). Accessible Telemedicine Diagnostics with ELISA in a 3D Printed Pipette Tip. Anal. Chem..

[34] Surface Chemistry Modification. https://harrickplasma.com/surface-chemistry/.

[35] Solidworks Solidworks 3D CAD. https://www.solidworks.com/product/solidworks-3d-cad.

[36] Phrozen Sonic Mighty 8K Resin 3D Printer. https://phrozen3d.com/products/sonic-mighty-8k.

[37] Conjure Rigid. https://www.chitusystems.com/products/conjure-rigid-clear-3d-printing-resin?variant=44530826608876.

[38] Shakeri A., Jarad N.A., Terryberry J., Khan S., Leung A., Chen S., Didar T.F. (2020). Antibody Micropatterned Lubricant-Infused Biosensors Enable Sub-Picogram Immunofluorescence Detection of Interleukin 6 in Human Whole Plasma. Small.

[39] Coad B.R., Vasilev K., Diener K.R., Hayball J.D., Short R.D., Griesser H.J. (2012). Immobilized Streptavidin Gradients as Bioconjugation Platforms. Langmuir.

[40] Human/Mouse/Rat Activin A Quantikine ELISA Kit (DAC00B) by R&D Systems, Part of Bio-Techne. https://www.bio-techne.com/p/elisa-kits/human-mouse-rat-activin-a-quantikine-elisa-kit_dac00b.

[41] Pawde S.M., Deshmukh K. (2009). Surface Characterization of Air Plasma Treated Poly Vinylidene Fluoride and Poly Methyl Methacrylate Films. Polym. Eng. Sci..

[42] Abdel–Fattah E. (2019). Surface Activation of Poly(Methyl Methacrylate) with Atmospheric Pressure Ar + H_2_O Plasma. Coatings.

[43] Rymuszka D., Terpiłowski K., Hołysz L., Chibowski E. (2015). Changes in Surface Properties of Polymethylmethacrylate (PMMA) Treated with Air Plasma. Ann. UMCS Chem..

