# Supplementary material for: A Facile Surface Modification Strategy for Antibody Immobilization on 3D-Printed Surfaces
Source: Biosensors (Basel). 2025 Mar 25;15(4):211. doi: 10.3390/bios15040211 (PMC12024930; doi:10.3390/bios15040211)
Supplement: Supplementary file 1 [file biosensors-15-00211-s001.zip › biosensors-3480215-supplementary.pdf]

Supporting Information

A Facile Surface Modification Strategy for Antibody Immobilization on 3D Printed Surfaces

Brandi Binkley [1] and Peng Li[1, *]

[1] Department of Chemistry, West Virginia University; bb00011@mix.wvu.edu
* Correspondence: peng.li@mail.wvu.edu

Table of contents:

Table S1. Data tables for the calibration curves in Figure 2 (top) medium sized wells (bottom) large sized wells.

Table S2. Data tables for the calibration curves in Figure 3 (top) silane modification (bottom) passive adsorption.

Table S3. Data table for the small well's calibration curve in Figure 4.

Table S4. Data table for the channel device calibration curve in Figure 5.

Table S5. Data tables for the calibration curves in Figure 6 (top) commercial wells (bottom) 3D printed wells.

Figure S1. Device design schematic and dimensions for 3D printed small wells.

Figure S2. Device design schematic and dimensions for 3D printed large wells.

Figure S3. Device design schematic and dimensions for 3D printed medium wells.

Figure S4. Device design schematic and dimensions for 3D printed channel device.

Figure S5. Contact angle study of a 20-microliter water droplet after different air plasma treatment lengths.

Table S1. Data tables for the calibration curves in Figure 2 (top) medium sized wells (bottom) large sized wells.

Activin A Concentration (pg/mL)	Average Fluorescence Intensity	Standard Deviation
62.5 pg/mL	583.76	137.4
125 pg/mL	864.88	53.1
250 pg/mL	1710.49	164.0
500 pg/mL	3138.28	125.5

Activin A Concentration (pg/mL)	Average Fluorescence Intensity	Standard Deviation
125 pg/mL	1599.30	182.6
250 pg/mL	3559.29	192.2
500 pg/mL	6097.72	526.5
1000 pg/mL	9413.36	283.2

Table S2. Data tables for the calibration curves in Figure 3 (top) silane modification (bottom) passive adsorption.

Activin A Concentration (pg/mL)	Average Fluorescence Intensity	Standard Deviation
62.5 pg/mL	583.76	137.4
125 pg/mL	864.88	53.1
250 pg/mL	1710.49	164.0
500 pg/mL	3138.28	125.5

Activin A Concentration (pg/mL)	Average Fluorescence Intensity	Standard Deviation
62.5 pg/mL	290.70	32.7
125 pg/mL	402.76	54.6
250 pg/mL	533.84	63.0
500 pg/mL	757.16	9.7

Table S3. Data table for the small well's calibration curve in Figure 4.

Activin A Concentration (pg/mL)	Average Fluorescence Intensity	Standard Deviation
125 pg/mL	834.58	82.7
250 pg/mL	2384.07	466.0
500 pg/mL	3528.10	417.6
1000 pg/mL	5804.40	848.8

Table S4. Data table for the channel device calibration curve in Figure 5.

Activin A Concentration (pg/mL)	Average Fluorescence Intensity	Standard Deviation
125 pg/mL	190.93	8.5
250 pg/mL	263.26	13.7
500 pg/mL	486.10	28.1
1000 pg/mL	1091.97	71.5

Table S5. Data tables for the calibration curves in Figure 6 (**top**) commercial wells (**bottom**) 3D printed wells.

Activin A Concentration (pg/mL)	Average Fluorescence Intensity	Standard Deviation
125 pg/mL	1278.47	359.4
250 pg/mL	2630.93	425.8
375 pg/mL	3455.22	428.5
500 pg/mL	4129.50	891.6
1000 pg/mL	7506.42	708.3

Activin A Concentration (pg/mL)	Average Fluorescence Intensity	Standard Deviation
125 pg/mL	1599.30	182.6
250 pg/mL	3559.29	192.2
375 pg/mL	4910.17	579.9
500 pg/mL	6097.72	526.5
1000 pg/mL	9413.36	283.2

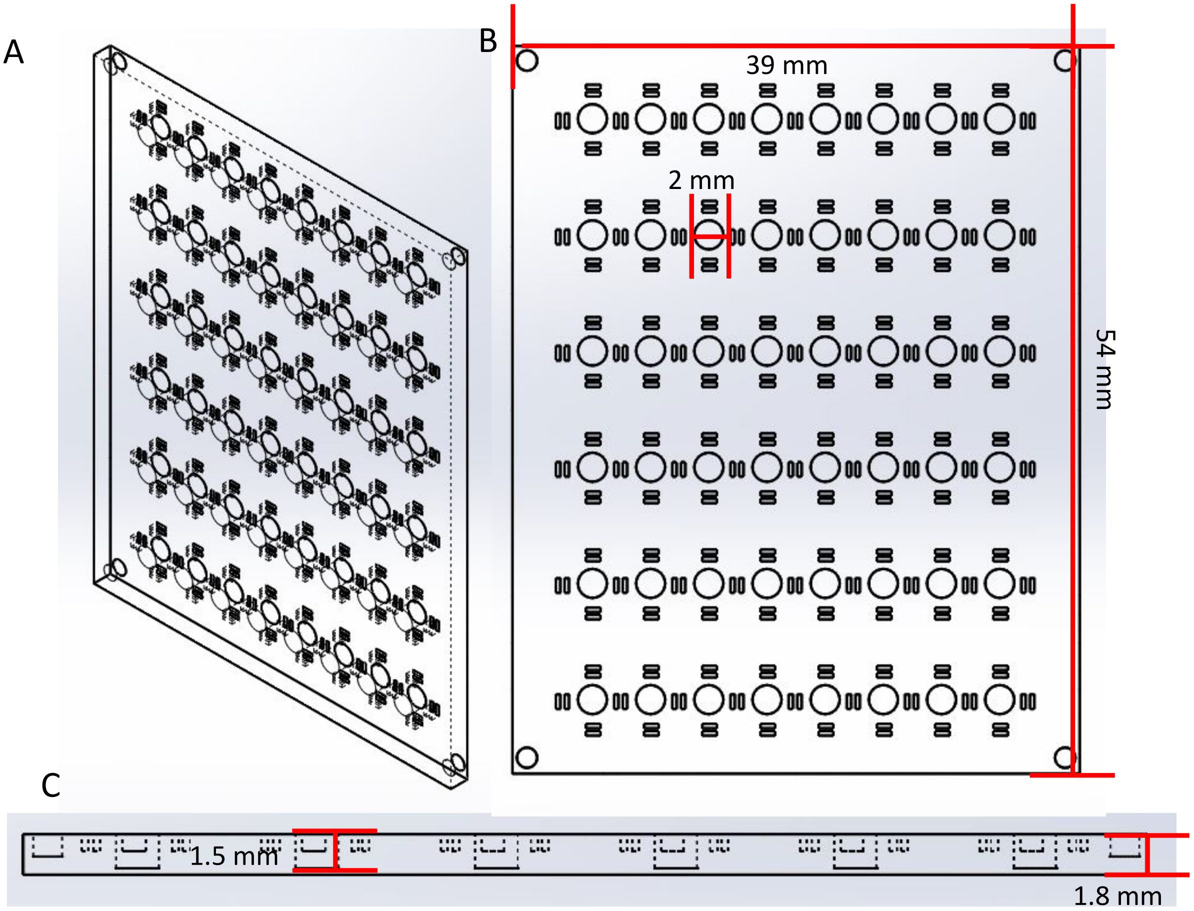

Figure S1. Device design schematic and dimensions for 3D printed small wells (**A**) angled view (**B**) top view (**C**) side view.

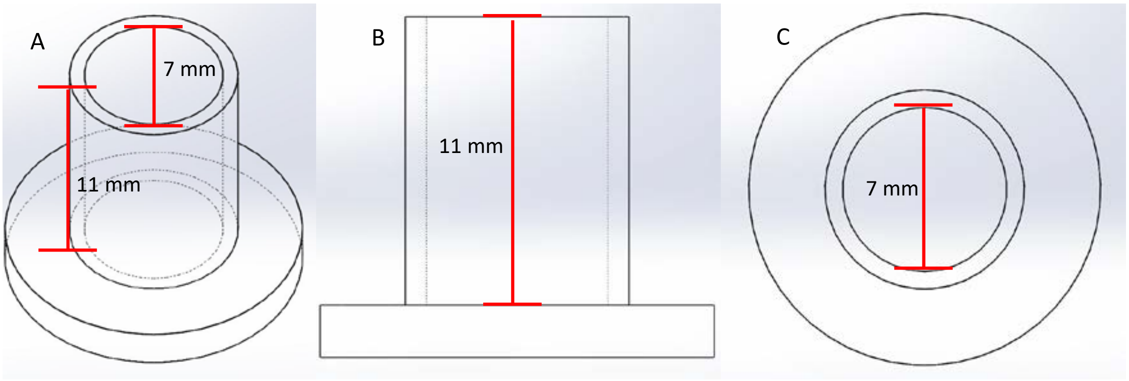

Figure S2. Device design schematic and dimensions for 3D printed large wells (**A**) angled view (**B**) side view (**C**) top view.

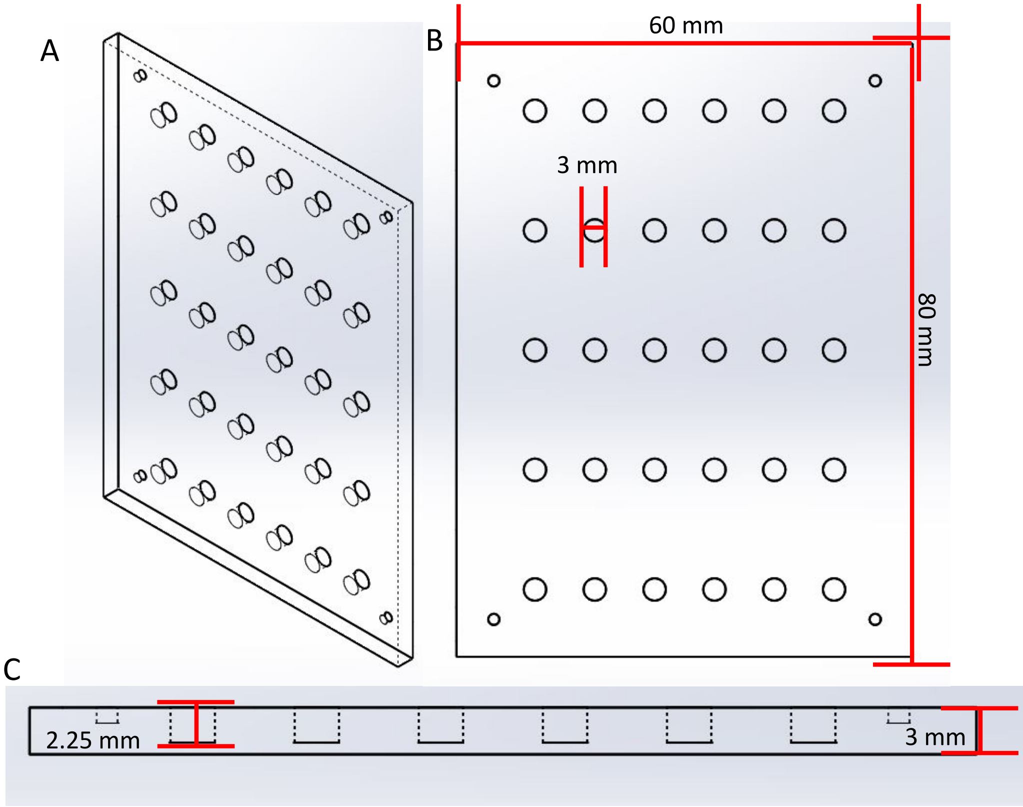

Figure S3. Device design schematic and dimensions for 3D printed medium wells. (**A**) angled view (**B**) top view (**C**) side view.

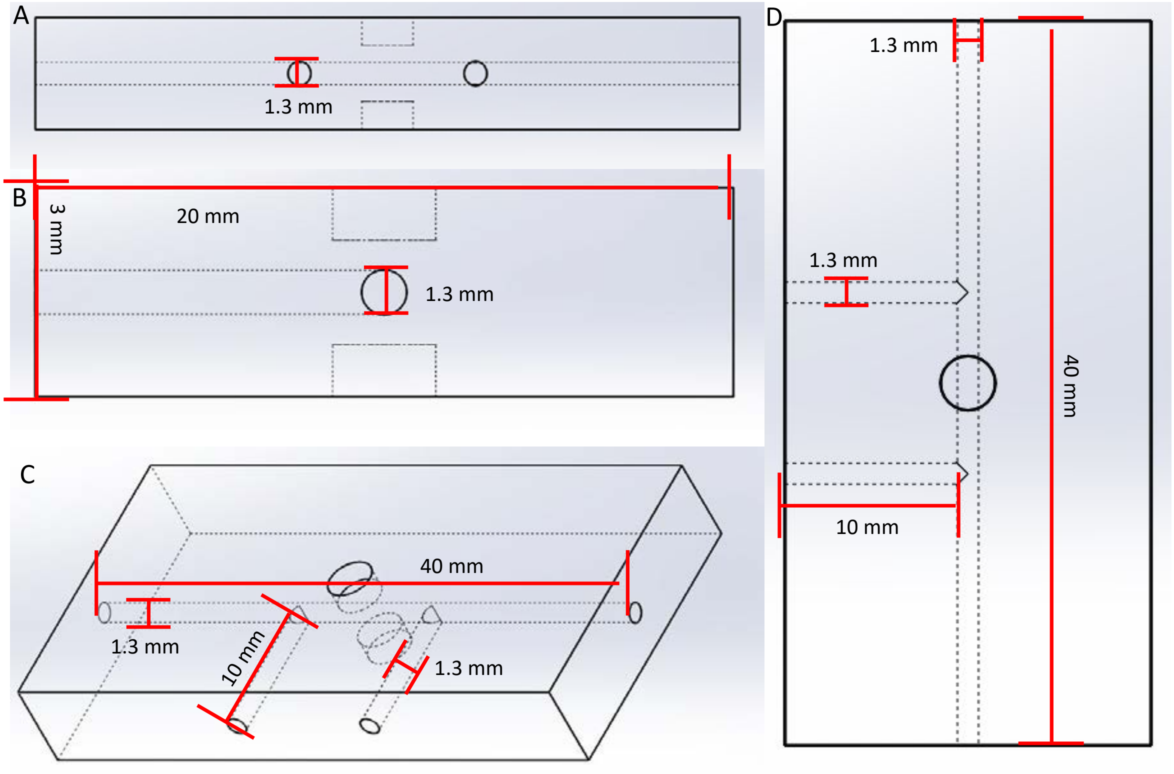

Figure S4. Device design schematic and dimensions for 3D printed channel device. (**A**) long side view (**B**) short side view (**C**) angled view (**D**) top view.

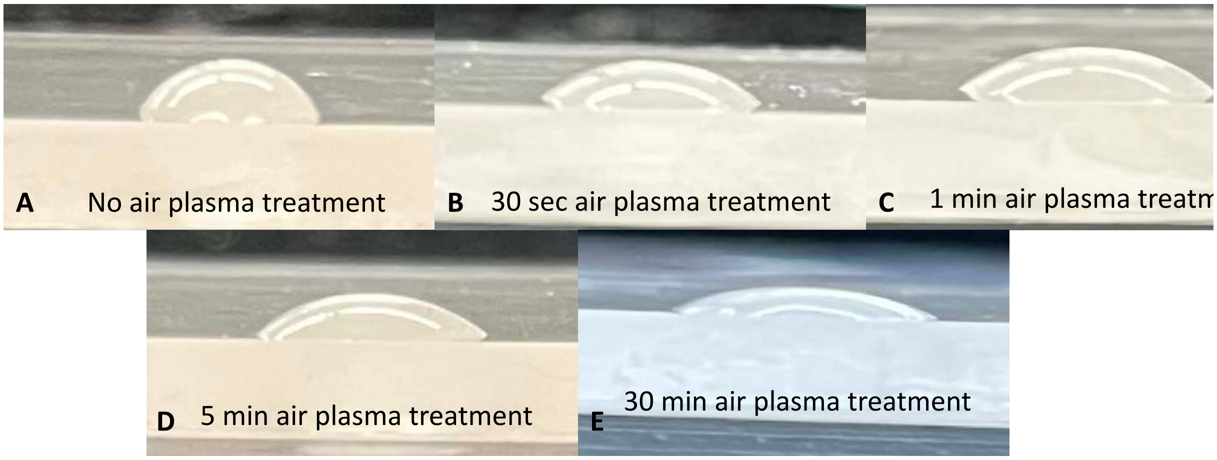

Figure S5. Contact angle study of a 20-microliter water droplet after different air plasma treatment lengths. (**A**) no air plasma treatment (**B**) 30 s (**C**) 1 min (**D**) 5 min (**E**) 30 min.
